# miR-203 drives breast cancer cell differentiation

**DOI:** 10.1186/s13058-023-01690-9

**Published:** 2023-08-04

**Authors:** Nuria G. Martínez-Illescas, Silvia Leal, Patricia González, Osvaldo Graña-Castro, Juan José Muñoz-Oliveira, Alfonso Cortés-Peña, María Gómez-Gil, Zaira Vega, Verónica Neva, Andrea Romero, Miguel Quintela-Fandino, Eva Ciruelos, Consuelo Sanz, Sofía Aragón, Leisy Sotolongo, Sara Jiménez, Eduardo Caleiras, Francisca Mulero, Cristina Sánchez, Marcos Malumbres, María Salazar-Roa

**Affiliations:** 1 Department of Biochemistry and Molecular Biology, School of Biology, Complutense University, Madrid, Spain; 2 Breast and Gynecologic Cancer Group, Research Institute i+12, Madrid, Spain; 3 Cell Division and Cancer Group, Molecular Oncology Program, Spanish National Cancer Research Centre (CNIO), Madrid, Spain; 4 Molecular Imaging Unit, CNIO, Madrid, Spain; 5 Histopathology Unit, CNIO, Madrid, Spain; 6 Bioinformatics Unit, CNIO, Madrid, Spain; 7 Department of Basic Medical Sciences, Institute of Applied Molecular Medicine (IMMA-Nemesio Díez), San Pablo-CEU University, Madrid, Spain; 8 Flow Cytometry and Fluorescence Microscopy Unit (CAI), Complutense University, Madrid, Spain; 9 Breast Cancer Clinical Research Unit, Clinical Research Program, CNIO, Madrid, Spain; 10 Hospital 12 de Octubre, Madrid, Spain; 11 Cancer Cell Cycle Group, Vall d’Hebron Institute of Oncology (VHIO), Barcelona, Spain; 12 ICREA, Passeig Lluís Companys 23, Barcelona, Spain

## Abstract

A hallmark of many malignant tumors is dedifferentiated (immature) cells bearing slight or no resemblance to the normal cells from which the cancer originated. Tumor dedifferentiated cells exhibit a higher capacity to survive to chemo and radiotherapies and have the ability to incite tumor relapse. Inducing cancer cell differentiation would abolish their self-renewal and invasive capacity and could be combined with the current standard of care, especially in poorly differentiated and aggressive tumors (with worst prognosis). However, differentiation therapy is still in its early stages and the intrinsic complexity of solid tumor heterogeneity demands innovative approaches in order to be efficiently translated into the clinic. We demonstrate here that microRNA 203, a potent driver of differentiation in pluripotent stem cells (ESCs and iPSCs), promotes the differentiation of mammary gland tumor cells. Combining mouse in vivo approaches and both mouse and human-derived tridimensional organoid cultures, we report that miR-203 influences the self-renewal capacity, plasticity and differentiation potential of breast cancer cells and prevents tumor cell growth in vivo. Our work sheds light on differentiation-based antitumor therapies and offers miR-203 as a promising tool for directly confronting the tumor-maintaining and regeneration capability of cancer cells.

**Supplementary Information:**

The online version contains supplementary material available at 10.1186/s13058-023-01690-9.

## Introduction

Solid tumors are heterogeneous in their cell composition, with a small subset of tumor cells sharing certain biological and molecular properties with tissue-specific stem cells (SC) [1–3]. This cell population has long-term renewal potential, supporting a proliferation hierarchy among cancer cells. In some tumors, the genes and signaling pathways that regulate normal SC roles also function as oncogenes or regulate tumor maintenance and progression [4–16]. One of the best-characterized examples is WNT–β-catenin signaling, which is essential for the maintenance and proliferation of SCs [17]. Importantly, this pathway is frequently mutated in colorectal cancers [18] and is required to sustain tumor growth and progression in several different types of cancers, including colorectal cancer, leukemia and skin basal cell carcinoma [19]. Consistent with the reprogramming of tumor cells into an embryonic-like fate, similarities between embryonic mammary SCs and the basal-like and HER2-positive breast cancer subtypes (which are less differentiated than other breast cancer subtypes) have also been described [20]. All those observations are consistent with the idea that, for tumor initiation, adult cells are required to undergo reprogramming to a progenitor-like fate.

Many of the current therapeutic strategies aimed at eliminating cancer cells involve treatment with standard antiproliferative chemotherapy, which often has limited benefits. The residual population of chemotherapy-resistant tumor cells capable of regenerating the disease is—at least by definition—enriched in cancer stem cells (CSCs) [1]. This fact has inspired the design of numerous antitumor therapies directly targeting the CSC niche, based on inducing their terminal differentiation. Indeed, the original idea of anti-CSC therapy arose in the 1970s and 1980s, from the observation that leukemic cells are blocked in an undifferentiated state. The use of all-trans retinoic acid induced terminal differentiation of leukemic cells [21]—and currently is the standard of care for the treatment of patients with acute promyelocytic leukemia. The success of all-trans retinoic acid therapy inspired other therapies that were based on inhibiting epigenetic regulators to induce cancer differentiation in multiple hematological malignancies [22] and the same mechanism also shows certain promise in solid tumors. Nevertheless, it is now established that even differentiated cells can be reprogrammed into stem-like cells, suggesting that cell state reprogramming is more common and occurs in more diverse cell types than previously thought [23, 24]. Indeed, this type of reprogramming can be used to re-establish stem-like hierarchies in tumors even after elimination of putative CSCs [25]. Therefore, eliminating unstable cells and also abrogating the mechanisms by which tumor cells gain cell state plasticity may be the most productive differentiation strategy. Such complexity makes the enticing therapeutic targeting of undifferentiated cancer cells still uncertain.

Novel players in carcinogenesis are microRNAs (miRNAs), which are epigenetically regulated but also control epigenetic events [26]. miRNAs comprise a class of small noncoding RNAs involved in posttranscriptional regulation of gene expression. miRNAs act by inhibiting translation of target mRNAs, and it is estimated that one-third of protein-coding mRNAs are subjected to regulation by miRNA. miRNA deregulation has been implicated in cancer development, and both oncogenic and tumor-suppressor miRNAs have been identified, many of which act through inhibition of translation of proteins controlling cell proliferation, survival and development [26].

Fuchs’ laboratory first described an in vivo role for microRNA 203 as a suppressor of stemness in developing epidermis [27]. Soon after that, we described miR-203 as a tumor suppressor in hematopoietic tumors. Our laboratory found that miR-203 expression was frequently silenced in mouse and human T and B cell malignancies through hypermethylation of its genomic region, and ABL1 and BCR-ABL1 fusion transcripts are indeed direct targets of miR-203-mediated translational repression [28]. The same year, a landmark paper from Massague’s group identified a set of eight microRNAs whose expression was inversely correlated with the metastatic potential of human breast cancer cell lines [29]. Though not studied further, miR-203 was among the eight miRNAs initially identified in that study. In the years since this report was published, miR-203 has been shown to regulate genes involved in crucial tumor pathways, such as signal transduction (BCR-ABL1), stemness (p63, BMI1), migration (LASP1, ASAP1), as well as known regulators of metastasis (SNAI1/2) among many others [30–39]. However, the capacity of miR-203 to fine-tune cancer cell differentiation remains uncertain and deserves a more focused research.

Recently, our laboratory has identified an unprecedented role of miR-203 modulating both reprogramming from somatic to pluripotent cells [40] and the differentiation capacity of stem cells [41]. Our data support the intriguing fact that a brief exposure to miR-203 blocks reprogramming to pluripotency while expanding the differentiation efficiency of stem cells. Such effects are mediated by direct or indirect targeting of the epigenetic landscape, making pluripotent cells more proficient for subsequent differentiation.

Given the obvious parallelisms between tumorigenesis and pluripotency [42–44], we evaluated the outcomes of miR-203 treatment on cancer cell differentiation. Using the classical MMTV-PyMT transgenic mice as a breast cancer model [45], we demonstrate here that miR-203-mediated effects on cellular reprogramming and cell differentiation can be advantageous in antitumor therapy. Combining in vivo approaches and their direct version on in vitro settings by tumor-derived organoids, we show that a brief exposure to miR-203 controls the self-renewal and proliferative capacity of breast cancer cells, attenuates migratory abilities and provokes a switch from a basal tumor phenotype to a more differentiated luminal-like status, similar to that observed in non-tumor cells.

## Results

### Different schedules of miR-203 treatment prevent tumor initiation, growth and metastasis in the MMTV-PyMT breast cancer mouse model

To easily manipulate miR-203 levels in vitro and in vivo, we generated a tetracycline-inducible knock-in model in which the miR-203-encoding sequence was inserted downstream of the type I collagen gene and expressed under the control of a tetracycline-responsive element [*ColA1* (miR-203) allele] in the presence of tetracycline reverse transactivator, expressed from the Rosa26 locus [Rosa26 (rtTA) allele] [41]. The treatment of *ColA1 (miR-203/miR-203); Rosa26 (rtTA/rtTA)*-derived cells with doxycycline (Dox) leads to a significant induction of miR-203 levels, only when exposed to Dox treatment [41].

To illustrate the role of miR-203 as an antitumor agent in vivo, we chose the PyMT breast cancer model for its close similarity to human breast cancer, exemplified by the fact that in these mice a gradual loss of steroid hormone receptors (estrogen and progesterone) and β1-integrin is associated with over-expression of ERBB2 and cyclin D1 in late-stage metastatic cancer [46]. In the Tg(MMTV-PyVT) model (also known as “MMTV-PyMT”), transgenic mice express the Polyoma Virus middle T (PyMT) antigen under the direction of the mouse mammary tumor virus (MMTV) promoter/enhancer [47]. Hemizygous MMTV-PyMT females develop palpable mammary tumors that metastasize to the lung and exhibit high penetrance and early onset of mammary cancer compared to other mammary tumor models. Tumor formation and progression in this murine model is notably similar to that observed in patients and is characterized by four stages: hyperplasia, adenoma/mammary intra-epithelial neoplasia, early carcinoma and late carcinoma. Therefore, we crossed our miR-203 inducible mice with the Tg(MMTV-PyVT) model, in order to generate an in vivo tool where easily fine-tune the miR-203 levels by Dox treatment in diet, at different time points during mammary tumor development.

**Fig. 1:**
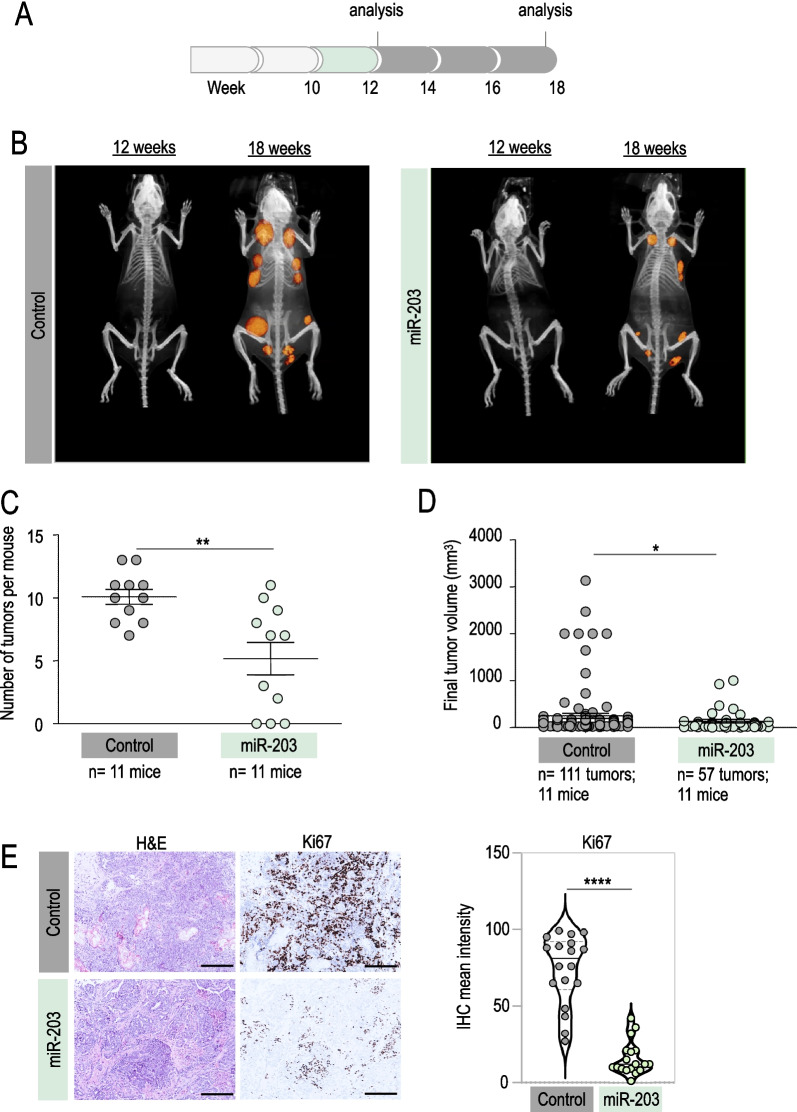
In vivo effects of miR-203 treatment on PyMT mice, started at tumor onset and sustained for two weeks. **A** Schematic of the doxycycline (Dox) treatment (in green) schedule in vivo, on *miR-203 wild-type* or *miR-203 knock-in*; *PyMT* mice, during two weeks from tumor onset (before the tumors are detected by micro-CT). **B** Representative micro-CT images of mice subjected to Dox treatment (in the figures, “control” indicates miR-203 wild-type; “miR-203” indicates knock-in mice), after Dox treatment (12 weeks of age) and at the endpoint (18 weeks of age). **C** Number of tumors per mouse at the endpoint, in control and miR-203-treated mice. **D** Final tumor volume of control and miR-203-treated mice. In **C**, **D**, data are represented as mean ± s.d. (Number of mice and total number of tumors per group are indicated in the figure.) **E** Left panel, Illustrative hematoxylin and eosin (H&E) and Ki67 immunohistochemistry (IHC) staining of control and miR-203-treated tumors at the endpoint. Right panel, Violin plot showing the quantification of Ki67 staining, six different fields from three independent tumor samples were analyzed. Scale bar, 500 µm. ****p < 0.0001; ** < 0.01; *p < 0.05 (Student’s t test)

**Fig. 2:**
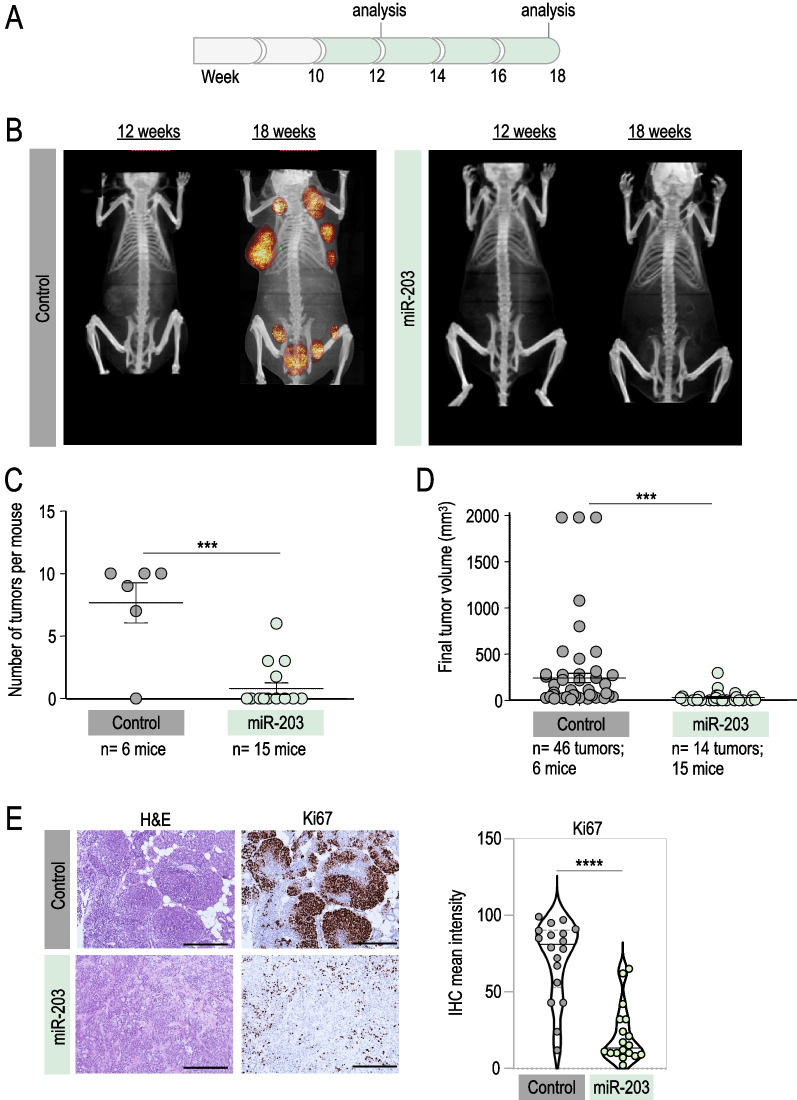
In vivo effects of miR-203 treatment on PyMT mice, started at tumor onset and sustained to the human endpoint. **A** Schematic of the Dox treatment (in green) schedule in vivo, on *miR-203 wild-type* or *miR-203 knock-in*; *PyMT* mice, from week 10 to the experimental endpoint. **B** Representative micro-CT images of mice subjected to the Dox treatment (in the figures, “control” indicates miR-203 wild-type; “miR-203” indicates knock-in mice), at 12 weeks of age and at the endpoint (18 weeks of age). **C** Number of tumors per mouse at the endpoint, in control and miR-203-treated mice. **D** Final tumor volume of control and miR-203-treated mice. In **C**, **D**, data are represented as mean ± s.d. (Number of mice and total number of tumors per group are indicated in the figure.) **E** Left panel, Representative H&E and Ki67 IHC staining of control and miR-203-treated tumors at the endpoint. Right panel, Violin plot showing the quantification of Ki67 staining, six different fields from three independent tumor samples were analyzed. Scale bar, 500 µm. ****p < 0.0001; ***p < 0.001 (Student’s t test)

**Fig. 3:**
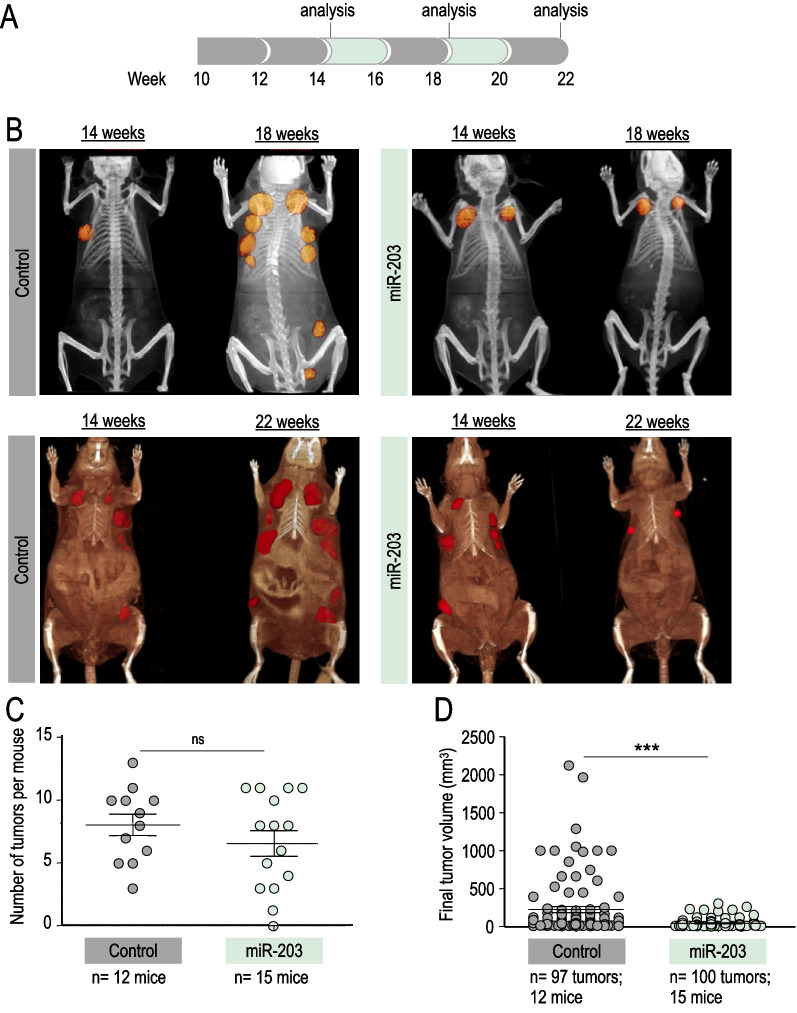
In vivo effects of miR-203 treatment on PyMT mice, started at tumor CT detection and administered every two weeks. **A** Schematic of the Dox treatment (in green) schedule in vivo, on *miR-203 wild-type* or *miR-203 knock-in*; *PyMT* mice, starting when tumors are found by micro-CT imaging (around week 14) to the endpoint, on alternating weeks. **B** Representative micro-CT images of mice subjected to the Dox treatment (in the figures, “control” indicates miR-203 wild-type; “miR-203” indicates knock-in mice) at tumor detection by micro-CT (14 weeks), four weeks later (18 weeks) and at the endpoint (22 weeks). **C** Number of tumors per mouse at the endpoint, in control and miR-203-treated mice. D, Final tumor volume of control and miR-203-treated mice. In **C**, **D**, data are represented as mean ± s.d. (Number of mice and total number of tumors per group are indicated in the figure.) ***p < 0.001; *n.s.* not statistically different (Student’s t test)

We dissected the in vivo antitumor effects of miR-203 by inducing its expression at different schedules: (i) starting at tumor onset and sustaining the treatment during two weeks (Fig. 1); (ii) starting at tumor onset and sustaining the treatment throughout the experiment, to the human experimental endpoint (Fig. 2); or (iii) starting once the tumors are established and under exponential growth, and treating every two weeks (Fig. 3). Tumors were followed by micro-CT, and the tumor volume was determined. The potential effects of miR-203 on metastasis incidence in the lungs were also evaluated by micro-CT throughout the three in vivo experiments, and by histopathology analysis of lung samples at the endpoint in all conditions tested.

As depicted in Fig. 1, we first treated *Tg (MMTV-PyMT); ColA1 (miR-203/miR-203); and Rosa26 (rtTA/rtTA)* mice (for short, *PyMT; miR-203 wild-type* or *PyMT; miR-203 knock-in*) with Dox during two weeks from tumor onset (week 10, as detected by histopathology analysis; Fig. 1A, Additional file 1: Fig. S1), followed by Dox withdrawal to the experimental human endpoint. The incidence of tumors per mice and the final tumor volume were significantly reduced in miR-203-treated compared to control mice (Fig. 1B–D). When the tumor samples were analyzed by immunohistochemistry at the end of the experiment, we found a down-regulation of the proliferation marker Ki67 in those tumors briefly treated with miR-203 in vivo, suggesting a less aggressive phenotype (Fig. 1E). No changes were detected in apoptosis, as assessed by the levels of cleaved caspase 3 (Additional file 1: Fig. S1).

As a next step, we maintained the Dox treatment from tumor onset to the experimental human endpoint (Fig. 2A, Additional file 1: Fig. S1). The miR-203-mediated antitumor effects were stronger in this case, since miRNA treatment blocked tumor onset almost completely (Fig. 2B, C). Indeed, very few tumors were found in the miR-203-treated group, and their final volume was notably reduced when compared to the control counterparts (Fig. 2C, D). The rare and small miR-203-exposed tumors we were able to analyze exhibited again a markedly reduced proliferation rate with no differences in apoptosis when compared to their control counterparts (Fig. 2E, Additional file 1: Fig. S1).

**Fig. 4:**
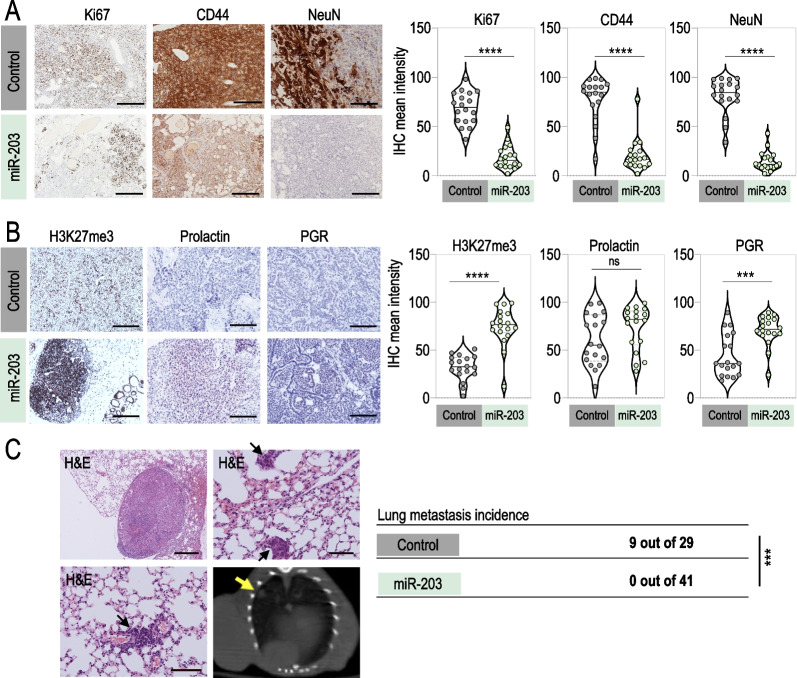
miR-203 exposure in vivo on PyMT mice alters the expression of stem-like and differentiation markers in mammary tumors and fully prevents lung metastasis. **A** Left panel, Representative images of IHC staining for Ki67 (to test proliferation), CD44 and NeuN (as stem-like cell markers) in control and miR-203-treated tumors, at the experimental endpoint and after exposure to Dox on alternating weeks from tumor detection by micro-CT, as indicated in Fig. 3A. Right panel, Violin plots showing the quantification of markers staining. **B** Left panel, Representative images of IHC staining for H3K27me3, prolactin and progesterone receptor (PGR), to test evidences of differentiation on control and miR-203-treated tumors as in (**A**). Right panel, Violin plots showing the quantification of markers staining. **C** Illustrative H&E staining of lung macro- and micro-metastasis, found in several control mice at the experimental human endpoint. Representative examples are shown, from the 9 metastasis cases identified throughout the three in vivo experiments (depicted in Figs. 1, 2, 3). As shown in the table, the overall incidence of metastasis was 31,03% in control mice *versus* 0% in miR-203-treated mice. The bottom right panel shows a representative micro-CT image, pointing to one evident macro-metastasis (yellow arrow) found in a control mouse. Scale bar, 500 µm. In violin plots, six different fields from three independent tumor samples were analyzed. ****p < 0.0001; ***p < 0.001; *n.s.* not statistically different (Student’s t test)

Finally, we tested an in vivo schedule where the treatment started once the tumors were detectable by micro-CT (around week 14) and was intermittently applied to mice, every two weeks, to the experimental endpoint (Fig. 3A). Of interest, this treatment schedule was significantly effective in terms of tumor growth control but the average of tumors detected per mouse was almost identical to the one observed in the control group (Fig. 3B–D), suggesting that, at this stage, tumor initiation capacity was recovered when the exposure to miR-203 was discontinuous. Since tumor initiation capacity falls—by definition—on dedifferentiated tumor cells, we tested whether well-established markers for dedifferentiation in cancer [48] had been altered by miR-203 treatment. CD44 and NeuN expression levels were notably reduced in those tumors exposed to miR-203 in vivo*,* as well as the proliferation marker Ki67 (Fig. 4A), while H3K27me3, prolactin and progesterone receptor, the three of them considered markers of maturation and differentiation [48–51], were induced on miR-203-treated tumors respect to the control counterparts (Fig. 4B). As an additional key observation, none of the miR-203-treated mice (*n* = 41) developed lung metastasis in any of the three in vivo experiments performed, compared to 31% incidence of lung metastasis in the control groups (*n* = 29; Fig. 4C).

Altogether, these observations demonstrate a beneficial role of miR-203 treatment not only to block proliferation and induce exhaustion of tumor growth capacity, but also to ameliorate metastasis incidence. With these data, it is tempting to speculate that miR-203 treatment has an impact on the cell renewal and plasticity of cancer cells.

### Brief exposure to miR-203 induces morphological and molecular changes suggestive of epithelial differentiation on PyMT mammary tumor-derived organoids

Recently, the culturing of mammary organoids in 3D artificial extracellular matrix (ECM) hydrogels has been shown as the most accurate approach especially for studying mammalian development, disease and stem cell behavior [52]. Previous studies demonstrated that organoids developed from breast tumors closely resemble the gene expression signature and heterogeneity of the tumor of origin, and even mammary branching morphogenesis is recapitulated in an organoid system by retaining its epithelial spatial organization [53–55]. Therefore, we decided to create an organoid platform in vitro, which helped us to interpret our observations in vivo and to characterize the antitumor effect of miR-203 with special focus on cancer differentiation. To generate the organoid cultures, we followed the same schedule depicted in Fig. 3, including this time a second control group of *PyMT; miR-203 knock-in* mice treated in vivo with vehicle. The outcome of both control groups was undistinguishable, corroborating that (i) Dox has no effect per se and (ii) the miR-203 inducible system is not leaky [41].

**Fig. 5:**
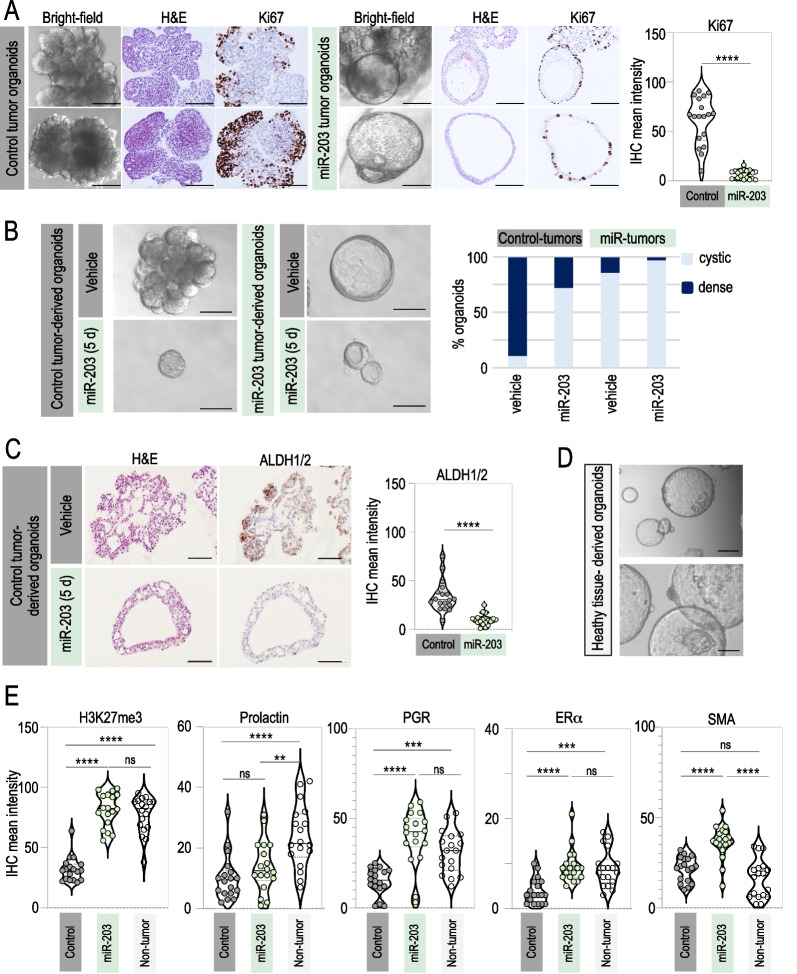
miR-203 transitory exposure promotes a morphological and molecular switch to epithelial differentiation on PyMT mammary tumor-derived organoids. **A** Left panel, Representative bright-field images and the corresponding H&E and Ki67 IHC staining of tumor-derived organoids (tumors from *miR-203 wild-type* or *miR-203 knock-in*; *PyMT* mice treated in vivo with Dox). Right panel, Violin plot showing the quantification of Ki67 staining. **B** Left panel, Representative bright-field images of tumor-derived organoids (tumors from *miR-203 knock-in*; *PyMT* mice treated in vivo either with vehicle or Dox), exposed in vitro to vehicle or miR-203 (Dox) during 5 days and followed by miR-203 withdrawal for 2 more weeks (indicated as “miR-203 5d” in the figure). Right panel, Quantification of the percentage of organoids exhibiting dense *versus* cystic (luminal-like) morphology in every condition tested. **C** Left panel, Representative images of H&E and ALDH1/2 IHC staining of control tumor-derived organoids, exposed to vehicle or miR-203 in vitro during 5 days and followed by miR-203 withdrawal, as in (**B**). Right panel, Violin plot showing the quantification of ALDH1/2 staining. **D** Representative bright-field images of healthy mammary gland-derived organoids (from *miR-203 wild-type*; *PyMT wild-type* mice). **E** Violin plots showing the quantification of H3K27me3, prolactin, progesterone receptor (PGR), estrogen receptor alpha (ERα) and smooth muscle actin (SMA) staining in control tumor-derived organoids (control), control tumor-derived organoids treated in vitro with miR-203 during 5 days (miR-203) and healthy mammary gland-derived organoids (non-tumor). Representative images are shown in Additional file 1: Fig. 2B. Scale bar 100 µm. In violin plots, six different fields from three independent tumor samples were analyzed. ****p < 0.0001; ***p < 0.001; ** < 0.01; *n.s.* not statistically different (Student’s t test for panels **A**–**C**; One-way ANOVA for **E**)

Interestingly, the morphology of control tumor-derived organoids was remarkably different to the one observed on miR-203-treated tumor-derived organoids. As depicted in Fig. 5A, the structure of control tumor-derived organoids was compacted, disorganized, dense and grape-shaped. However, miR-203-treated tumor-derived organoids were predominantly cystic and structured, suggestive of a luminal epithelium [54]. Moreover, immune-histology analysis revealed lower proliferative rates (Ki67 staining) in miR-203-treated tumor-derived organoids when compared to their control counterparts (Fig. 5A). When the control organoids (never exposed to Dox in vivo) were exposed to miR-203 in vitro for a short period of time (5 days, followed by miR-203 withdrawal for two more weeks), their morphology systematically changed in a gradual manner turning into hollow cysts (Fig. 5B, Additional file 1: Fig. S2A), showing again that miR-203 treatment boosts the cyst-forming ability of mammary epithelial cells. Of interest, such capacity has been attributed to ALDH-positive progenitors [56]. Accordingly, the expression levels of ALDH1/2 were notably diminished when the organoids were briefly exposed to miR-203 in vitro (Fig. 5C), suggesting the terminal differentiation of such progenitors. The cystic organoids exposed to miR-203 eventually collapsed (as denoted in the bright-field images of Fig. 5B and Additional file 1: Fig. S2A), while the control organoids were easily maintained in vitro for several passages during months.

Intriguingly, healthy mammary gland tissue-derived organoids exhibited a similar phenotype to the one observed in the miR-203-treated organoids: cystic, organized and morphologically luminal-like (Fig. 5D). The induced expression of the epigenetic marker H3K27me3, associated to differentiation [49, 57], was prominent in miR-203-treated tumor organoids and healthy mammary gland-derived organoids, when compared to the control tumor counterparts (Fig. 5E and Additional file 1: Fig. S2B). We further analyzed the expression of other markers linked to differentiation, such as prolactin, progesterone receptor (PGR), estrogen receptor alpha (ERα) and smooth muscle actin (SMA) [49], and both the healthy tissue-derived organoids and the miR-203-treated tumor organoids exhibited comparable staining for all the molecular markers tested (Fig. 5E and Additional file 1: Fig. S2B).

Since miR-203 induced morphological and molecular changes in the tumor-derived organoids suggestive of cancer cell differentiation, we next examined whether these changes were similar to the ones triggered by other well-known differentiation stimuli. Thus, we tested in our culture a defined epithelial differentiation media (detailed in methods section) and FGF2 treatment, described to induce branching morphogenesis [56, 58, 59]. The healthy tissue-derived organoids mostly presented a cystic morphology in any condition tested, with the exception of FGF2 treatment, which always induced the mammary trees typical of branching morphogenesis (Additional file 1: Fig. S2C). On the contrary, the tumor-derived organoids were more susceptible to treatment-induced changes: while mostly condensed and grape-shaped upon basic expansion media, the tumor-derived organoids shifted to a predominant cystic morphology, induced by epithelial differentiation media and particularly by miR-203 expression, suggesting that any of those treatments were boosting the cyst-forming ability of mammary epithelial cells. Interestingly, and as occurred with the healthy tissue-derived organoids, those tumor organoids cultured in the presence of FGF2 exhibited a prominent branching morphology, either treated or not with miR-203 (Additional file 1: Fig. S2C).

Altogether, these observations suggest that a short exposure to miR-203 favors mammary epithelial differentiation on tumor organoids, which also correlates with a direct detrimental effect of this microRNA on the propagation and expansion of the organoid culture.

### Brief exposure to miR-203 induces a basal-to-luminal switch on mouse PyMT mammary tumor-derived organoids

**Fig. 6:**
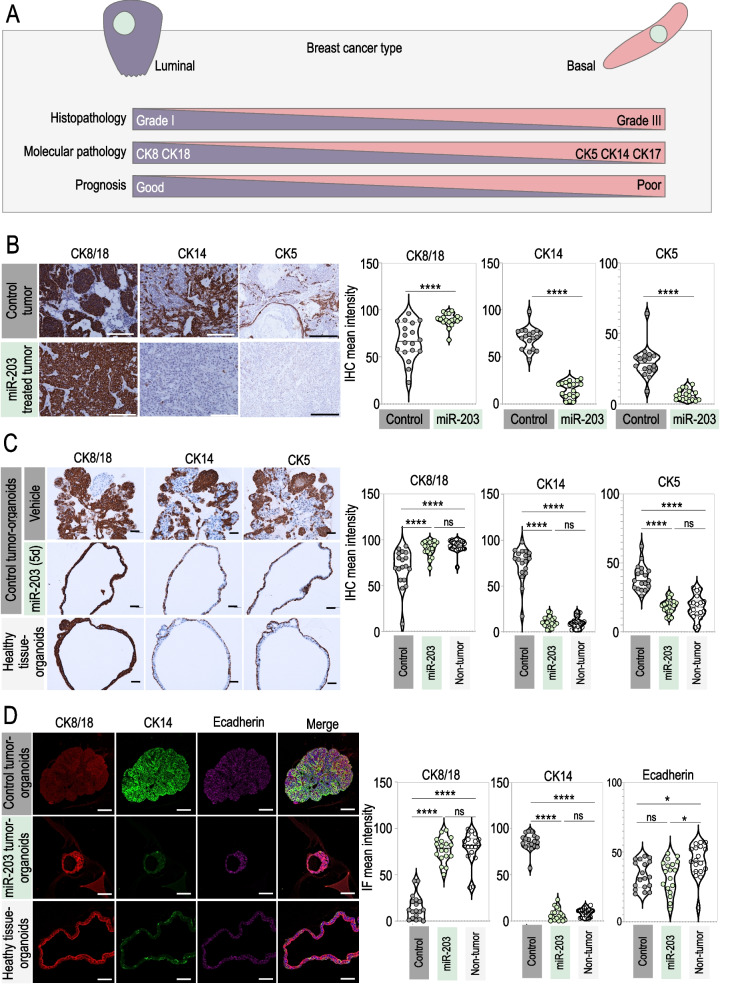
miR-203 transitory exposure induces a basal-to-luminal shift on mouse mammary tumor-derived organoids. **A** Schematic showing the correlation between cytokeratins expression, histopathological tumor grade, prognosis and breast cancer type. **B** Left panel, Illustrative detection of CK8/18, CK14 and CK5 by IHC in control tumors and miR-203-treated tumors at the experimental endpoint. Right panel, Violin plots showing the quantification of markers staining. The doxycycline schedule followed for this set of experiments is also the one indicated in Fig. 3A. **C** Left panel, Illustrative IHC images of staining for CK8/18, CK14 and CK5 in control tumor-derived organoids, control tumor-derived organoids treated in vitro with miR-203 during 5 days and healthy mammary gland-derived organoids. Right panel, Violin plots showing the quantification of markers staining. **D** Left panel, Detection of CK8/18 (red), CK14 (green) and E-cadherin (purple) by immunofluorescence in tumor-derived organoids, extracted from control tumors, miR-203-treated tumors or healthy mammary gland tissue samples. Right panel, Violin plots showing the quantification of markers staining. In **B**, **C** scale bar, 500 µm; in **D**: scale bar, 100 µm. In violin plots, six different fields from three independent tumor samples were analyzed. ****p < 0.0001; *p < 0.05; *n.s.* not statistically different (Student’s t test for panel B; One-way ANOVA for panels **C**, **D**)

In combination with other markers, cytokeratins (CK) have been used for a long time to determine the origin and grade of breast cancers. As represented in the schematic of Fig. 6A, cytokeratins 5, 14, and 17 are mostly associated to basal (and therefore poorly differentiated) tumors and poor patient prognosis, while cytokeratins 8 and 18 depict a luminal origin (and therefore highly differentiated status) and denote good patient prognosis [48, 57, 60–63]. Following those well-established histopathology correlations, we tested by immunohistochemistry CK5, CK14 and CK8/18 expression levels in control tumors *versus* miR-203-treated tumors. Importantly, CK5 and CK14 staining was markedly reduced, while CK8/18 expression levels were induced in miR-203-treated tumors when compared to the control ones (Fig. 6B) suggesting a basal-to-luminal switch. Accordingly, tumor-derived organoids exhibited expression levels for CK5, CK14 and CK8/18 comparable to their corresponding tumors of origin, while miR-203 short exposure in vitro reduced the expression levels of CK5 and CK14 and induced the expression of CK8/18 (Fig. 6C). Again, the staining in miR-203-exposed organoids was comparable to that in healthy tissue-derived organoids for all the CK markers tested (Fig. 6C).

We performed additional immunofluorescence experiments on miR-203-exposed tumor-derived organoids and the corresponding controls, to corroborate those observations. The upper images in Fig. 6D show a representative example of control tumor-derived organoids, exhibiting high levels of CK14 and low levels of CK8/18, while miR-203-treated tumor-derived organoids (middle images) shifted the cytokeratins expression profile, being the CK8/18 the most predominant and CK14 becoming much less represented, almost absent. Again, the healthy tissue-derived organoids (lower images) exhibited a similar phenotype to the one observed in the miR-203-treated tumor-derived organoids.

To further understand the mechanistic insights of the differentiation-based antitumor effects evoked by miR-203, we performed RNA sequencing of organoid samples, derived from healthy or tumor tissue, and exposed in vitro to miR-203 (during 5 days, followed by 14 days of miR-203 withdrawal) or epithelial differentiation media (Additional file 1: Fig. S3). Principal component analysis of those samples revealed a prominent effect of miR-203 treatment only on tumor-derived organoids. To some extent, the transcriptomic profile induced by miR-203 on the tumor organoids appeared to be parallel to that induced by the differentiation media, suggesting a partial similarity between both treatments (Additional file 1: Fig. S3A). On the other hand, the differentiation media notably altered—while miR-203 treatment did not significantly modify—the transcriptomic profile of healthy tissue samples (Additional file 1: Fig. S3A). When a specific signature for “Mammary Gland Development” was considered, we identified a divergence between non-tumor and tumor organoids, as expected. Of interest, miR-203 treatment partially reverted such differences only in tumor organoids, for genes involved in this particular signature and also in the sub-cluster “Mammary Gland Epithelial Differentiation” (Additional file 1: Fig. S3B, S3C). Enrichment plots for the “Mammary Gland Stem Cell (MaSC)” and “Mature Luminal Cell” signatures revealed a significant correlation between miR-203 treatment and the induction of genes characteristic of mature luminal cells, while no correlation was observed for MaSC genes (Additional file 1: Fig. S3D). When we interrogated the gene expression profile of the epithelial-to-mesenchymal transition (EMT), the miR-203 exposed samples showed a poor correlation, in contrast to those incubated in differentiation media (Additional file 1: Fig. S3D). Besides, the bulk RNA sequencing performed here supported our former observations, such as major alterations provoked by miR-203 brief exposure in the mRNA expression levels of cytokeratins, progenitor markers, EMT markers, differentiation markers and, interestingly, key cell cycle regulators such as Cdk1, among others (Additional file 1: Fig. S4 and Additional file 2: Table 1). Of interest, gene signatures for “Basal Cells” (as defined by two different data bases) were significantly down-regulated by miR-203 treatment (Additional file 1: Fig. S4A) as well as gene signatures for “Organ and Cell Development,” “Cell Migration and Motility,” “Cell Metabolism” and “Cell Cycle” (Additional file 1: Fig. S4B and Additional file 2: Table 1).

Altogether, these data on PyMT breast cancer organoids demonstrate that a brief exposure to miR-203, either in vitro or in vivo, induces a shift from a basal tumor phenotype to a more differentiated luminal epithelial status.

### Brief exposure to miR-203 induces a basal-to-luminal shift and reduces collective migration on patient-derived breast tumor organoids

**Fig. 7:**
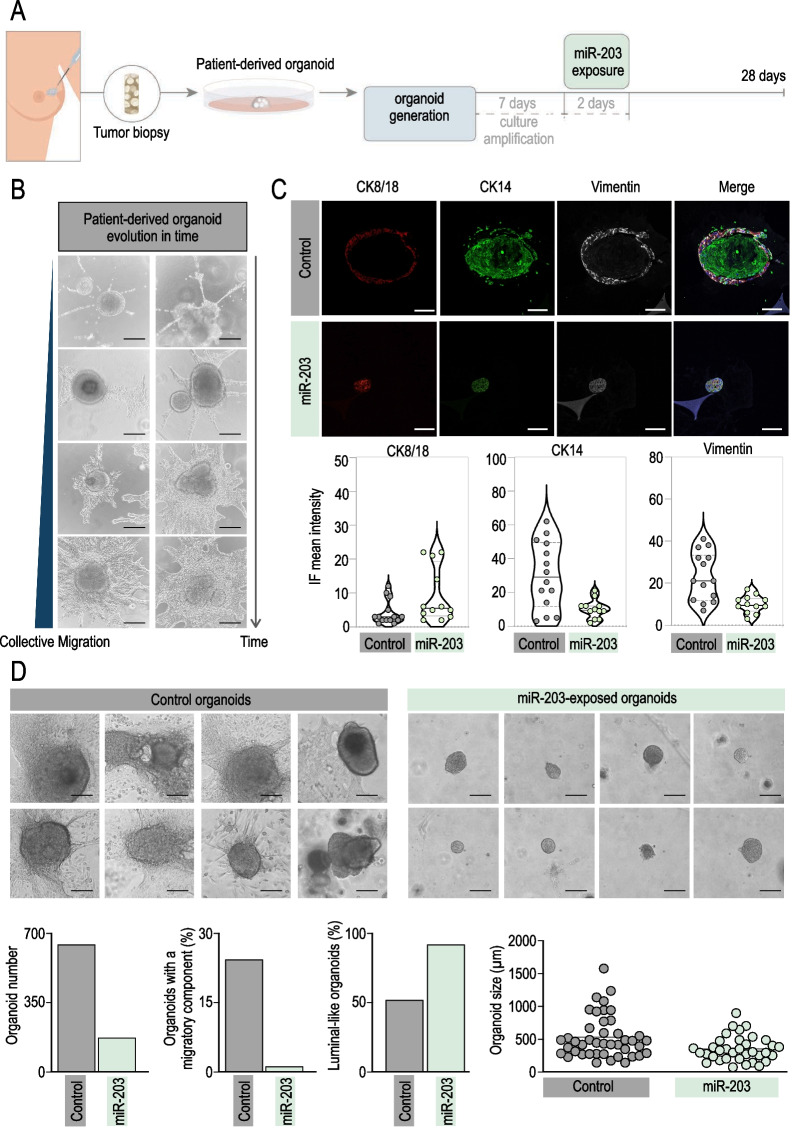
miR-203 transitory exposure induces a basal-to-luminal shift and reduces collective migration on patient-derived breast tumor organoids. **A** Schematic showing the experimental procedures followed for patient-derived tumor processing, organoid culture establishment and miR-203 mimics transient transfection. **B** Representative bright-field images showing the progressive collective cell migration projected from the 3D patient-derived organoids along time. **C** Upper panel, Detection of CK8/18 (red), CK14 (green) and vimentin (white) by immunofluorescence in patient tumor-derived organoids, transiently exposed or not to miR-203 mimics in vitro. Lower panel, Violin plots showing the quantification of markers staining, six/seven different fields from two independent tumor samples were analyzed. **D** Upper panels, Representative bright-field images of patient-derived organoids, control *versus* miR-203 briefly exposed, denoting the morphological differences in complexity, size and migration upon miR-203 treatment. Lower panels: quantification of the total number of organoids, percentage of organoids exhibiting collective migration, percentage of organoids with luminal-like morphology and organoid size, of control *versus* miR-203 briefly exposed patient-derived organoids; *n* = 3 technical replications from each 2 biological samples (2 independent biopsies). Receptor status of the two patient samples shown is the following: (1) 80% ER; 60% PR; 18% Ki67 index; and grade 1 HER2. (2) 80% ER; 80% PR; 15% Ki67 index; and grade 2 HER2. Both patients were enrolled in a clinical trial. In **B**-**D**: Scale bar, 100 µm

To explore the therapeutic potential of miR-203 in humans, we evaluated its effects on breast cancer patient-derived organoids. Figure 7A shows a schematic of the procedures followed with patient-derived organoids and the temporal line of the experimental settings. As depicted, after 7 days of culture establishment and organoid amplification, patient-derived 3D cultures were transiently transfected with synthetic miR-203 mimics, followed by miR-203 withdrawal for three additional weeks, when the analysis was performed. We observed the 3D cultures under the bright-field microscope along the experiments to evaluate every potential morphological alteration induced by the short exposure to miR-203. Soon after organoid establishment, we systematically observed the formation of cell spire protrusions only in control organoids (Fig. 7B). Elongated cells emerged from the organoid edges, and whenever made contact with a solid surface (i.e., the plastic or glass well bottom), they attached to it and gradually occupied the surrounding area forming a bi-dimensional layer below and beyond the tridimensional organoids (Fig. 7B). It is reasonable to speculate that these cells undergo collective migration. It has been described that tumor cells may experience a partial EMT with their cell–cell connections remaining intact and thereby migrate as a cohesive group [64]. The leader cells use similar mechanisms as migrating single cells to polarize, protrude, invade and adhere to stromal matrix, and they are generally more organized and efficient in direct invasion than the individual cells [64–66]. Molecularly, we detected a reproducible pattern of front-rear polarity for the expression of cytokeratins and the EMT marker vimentin (Fig. 7C): In control organoids, CK14 and vimentin appeared highly expressed within the cells conforming the external organoid layer and those attaching to the plate surface, while CK8/18 was almost undetectable. Of interest, a short and transient exposure to miR-203 blocked the cell projections and migration from the organoids (images and quantifications in Fig. 7D), reduced the expression of CK14 and vimentin to almost undetectable levels and, in turn, stimulated the expression of CK8/18 (Fig. 7C). Of importance, not only collective migration was dropped by the exposure to miR-203 but also the total number of organoids, their complexity and their size were notably reduced, while the proportion of luminal-like organoids in the culture was significantly augmented (Fig. 7D).

Altogether, the data presented here corroborate, in patient-derived samples, the potential of miR-203 as a cancer differentiation driver of breast cancer cells, with possible implications in cancer therapy.

## Discussion

Cancer has been broadly interpreted as a caricature of normal tissue development. Cellular programs regulating tissue plasticity, self-renewal and expansion are exquisitely orchestrated under physiological conditions. However, aberrant tumor mechanisms unbalance this coordinated cell plasticity and give rise to immature or dedifferentiated tumor cells. Indeed, it is now widely accepted that, for tumor initiation, adult cells experience reprogramming to a progenitor-like fate [67]. Thus, tumor dedifferentiation supports cancer progression, relapse and metastasis. Traditional chemo- and radiotherapy generally involves the elimination of proliferating tumor cells. Instead, the differentiation therapies offer the possibility of coaxing cancer cells into becoming normal cells, reactivating the endogenous differentiation programs to resume maturation. Cancer differentiation approaches are still evolving and require novel methodologies to reach efficient therapies. Trusting this general believe, we decided to examine the antitumor effects of miR-203 from a cancer differentiation perspective. This microRNA has been recently described by our group to fine-tune the critical balance between reprogramming, stemness and differentiation programs: miR-203 blocks somatic-to-pluripotency reprogramming [40], while potentiates differentiation of stem cells to a mature and terminal state [41]. We hypothesized that such effects could be applied to cancer differentiation and therefore would point to miR-203 as a promising tool for differentiation-based antitumor therapy.

In vivo, we tested different treatment schedules, trying to understand the consequences of treatment intermittency. Interestingly, only when mice were exposed to miR-203 from tumor onset to the end of the experiment, tumor initiation and growth were completely prevented. When miR-203 treatment was intermittent, we noticed a significant control on tumor growth and a considerable delay in tumor initiation, while we did not completely avoid the latter. In this line, recent advances on stem cell biology have demonstrated that stem cell plasticity represents one of the major therapeutic challenges for differentiation therapies. Several studies have provided evidence that both CSCs and non-CSCs are plastic and capable of undergoing phenotypic transitions in response to appropriate stimuli. This notion was for instance exemplified by a study in which cell populations displaying stem cell-, basal- or luminal-like phenotypes were isolated from breast cancer cell lines [68]. In vitro, all three subpopulations were able to generate cells of the other two phenotypes. This phenotypic inter-conversion was stochastic and not determined by the cell phenotype of origin. Thus, it is accepted now that CSC and non-CSC states are not hardwired: considering that plasticity may be in tumor cells as extensive as it is in healthy tissues, CSCs would be always recreated. This fact could explain the successful outcome when miR-203 treatment was uninterrupted (Fig. 2), theoretically capturing any newly formed CSC. However, the intermittency in miR-203 exposure would tentatively allow the undifferentiated population to restore, favoring then the tumor initiation process. We interpreted that miR-203 exposure was able to maintain the stem-like capacity delimited and therefore tumor growth under control in any regimen tested. One of the key results in vivo, denoting the strong effect of miR-203 on tumor differentiation, was the lack of metastasis (suggestive of detrimental migratory and invasive capacity) detected on those mice exposed to miR-203 in any of the treatment schedules tested, while their control counterparts experience a metastasis incidence of 31%. Of interest, in vitro cell cultures derived from patient biopsies exhibited a polarized and migratory cell population that was completely abolished by miR-203 treatment. Together, those two interesting observations prompt us to speculate that miR-203 impact on cancer cell differentiation plays a role in invasion and metastasis.

Inspired by the natural development of the mammary epithelium, Hans Clevers and collaborators established culture protocols that allow the generation and long-term expansion of three-dimensional mammary gland- or tumor-derived organoids [69, 70]. We efficiently produced organoids from the mouse tumors or mammary glands, recapitulating the tridimensional architecture and the molecular features of the source tissue, and thus allowing a deeper analysis of the cancer stem cell behavior in vitro.

Our first striking observation was the remarkable differences on organoid morphology upon miR-203 treatment: The structure of control tumor-derived organoids was notably compacted, disorganized, dense and in most cases, grape-shaped, while the miR-203-treated tumor-derived organoids were predominantly cystic, suggesting an epithelial luminal origin. Of interest, the cystic morphology was reproduced any time the tumor-derived organoids were yielded to transient miR-203 treatment in vitro.

This observation is not surprising if we consider that the adult virgin mammary gland is a highly organized tree-like structure, formed by ducts with hollowed lumen. It has been widely demonstrated that a controlled and delimited induction of apoptosis is crucial for clearing the lumen in terminal end buds during puberty. Apparently, the differentiation process observed in the organoids implies a similar process, spatially and temporally organized, where the lumen is shaped and the cells distribute adjacent the newly formed cystic structure. Moreover, those cystic organoids resulting from miR-203 exposure, either in vivo or in vitro, collapsed after a few passages, contrary to the control organoids never treated with the microRNA, which were long-term maintained in culture, as published before [54, 69, 70]. The short lifespan of organoids exposed to miR-203 clearly pointed to an exhaustion of the self-renewal capacity of the culture—possibly accompanied by an increased cell death index—and implied a direct detrimental effect by this miRNA on the propagation and expansion of the organoids. The induction of differentiation comprises a plethora of signals, cellular-transduced and ultimately translated into a complex combination of responses. Accordingly, Ki67, CK5, CK14 and ALDH1/2 levels were diminished in those miR-203-exposed organoids, while markers such as CK8/18 were induced, implying a shift from dedifferentiated basal to a more differentiated luminal-like phenotype.

One of the questions we have not answered in this work is the cellular origin of miR-203. Our data in organoids suggest a cell-autonomous effect on mammary gland cells, and indeed denote a clear luminal character. It has been published that miR-203 is activated during luminal epithelial differentiation and this pattern is observed in the murine mammary hierarchy [71]. Also, in a paper where microRNAs signatures of distinct mammary epithelial cell types were analyzed, miR-203 was found as delimited to luminal cells again, targeting basal-restricted genes [72]. Although a deeper analysis should be done to elucidate the cellular subpopulation responsible for such observed effects, it is tempting to speculate that-at least in our model system- the over-expression of miR-203 in luminal mammary gland cells exacerbates a naturally designed differentiation program in this cell subtype.

Of interest, the healthy mammary gland tissue-derived organoids exhibited a very similar phenotype to the one observed in the miR-203-treated tumor organoids (Figs. 5D, 5E, 6C, D and Additional file 1: Fig. S2B, S2C). Several markers associated to differentiation were found to be more expressed in healthy tissue-derived as well as in miR-203-exposed tumor organoids.

Deepening into the differentiation concept, we tested in our organoid platform some previously defined differentiation conditions, such as FGF2-mediated induction of mammary branching or a culture medium described for mammary epithelial cell differentiation. The tumor-derived organoids were mostly condensed and grape-shaped upon basic expansion media, and shifted to a predominantly hollow cysts morphology when exposed to the epithelial differentiation media or (even more dramatically) to miR-203, suggesting that any of those treatments were boosting the cyst-forming ability of mammary epithelial cells. As expected, FGF2 treatment always induced the mammary trees typical of branching morphogenesis [56] either on non-tumor or tumor organoids, exposed or not to miR-203. The intriguing fact that, upon FGF2 treatment, miR-203 does not induce epithelial cystic formations but instead favors the branching constructions, was in concordance with our previous works and others [40, 41, 73] and suggests that, submitted to a strong differentiation scenario, miR-203 acts always as a differentiation enhancer and not as a reprogramming inducer, and also reinforces its role as a regulator of branching morphogenesis and basement remodeling.

When the transcriptomic profiles of those organoids were tested by RNA sequencing, we noticed a remarkable impact of miR-203 treatment on the differentiation of tumor-derived organoids, while the healthy tissue-derived organoids showed very little alterations when exposed to miR-203. These data were in concordance with the phenotypically observed modifications incited by miR-203: Whereas miR-203 exposure completely shifted the shape of tumor organoids, no notable changes were induced on non-tumor organoids. This suggests a fascinating differential impact of this microRNA on tumor and non-tumor tissues that deserves to be further explored. Upcoming works based on single cell analysis would define the mechanistic insights of miR-203 as a tumor differentiation agent, outlining its influence on the transcriptomic, genomic and epigenetic landscapes of the different cell subtypes.

Several targets have been defined for miR-203 in cancer, conferring mostly a role as tumor suppressor [30], or eventually, as tumor promoter [74]. This apparent discrepancy could be explained by the distinct models and in vitro systems used in such studies, and how the context strongly influences the microRNA target dependencies and outcomes. Our work highlights how dedifferentiation influences tumorigenesis, and in the context of breast cancer, the potential therapeutic advantages of targeting stem-like basal cells and differentiating them into luminal cells. Thus, the data presented here not only confirm the antitumor effects mediated by miR-203, but also particularly denote its influence on cancer differentiation, both in murine and patient-derived samples. This work undoubtedly opens new perspectives on the potential therapeutic applications of miR-203 in cancer.

## Methods

### Animal models and procedures

Animal experimentation was performed according to protocols approved by the CNIO-ISCIII and the UCM Ethics Committee for Research and Animal Welfare (CEIyBA) and Madrid Regional Government, according to European official regulations. The miR-203 inducible model was generated by cloning a 482-bp genomic mmu-mir203 sequence into the pBS31 vector for recombination into the ColA1 locus in embryonic stem cells. The resulting knock-in allele [ColA1(miR-203)] was combined with a Rosa26-M2rtTA allele [Rosa26(rtTA)] for doxycycline-dependent induction as described previously [41]. PyMT [FVB/N‐Tg(MMTV‐PyVT)^634Mul/J^] mice were kindly provided by Miguel Quintela (CNIO, Spain). Mice were then crossed to obtain the Tg (MMTV-PyMT); Rosa26(rtTA); and ColA1(miR-203) strain, which has been used throughout this work. To induce miR-203 expression in vivo, doxycycline (Dox) was orally administered to mice in diet (Dox-delayed release pellets, from Jackson laboratories) following the different schedules indicated in Figs. 1, 2, 3. As control, Dox treatment was applied to Tg (MMTV-PyMT); miR-203 (+ / +) mice, which also served as an internal checkup of the Dox treatment itself.

Primers used for genotyping the PyMT transgene were 5′-GGAAGCAAGTACTTCACAAGG-3′ and 3′-GGAAAGTCACTAGGAGCAGGG-5′. Polymerase chain reaction conditions were as follows: 95 °C for 15 min; 94 °C for 30 s; 30 cycles at 59 °C for 45 s; 72 °C for 1 min; 72 °C for 10 min; and then soaking at 4 °C. PCR products are 336 bp (base pair) for wt allele, 438 bp for lox allele, 470 bp for cre allele and 557 bp for PyMT allele. All these animals were maintained in a mixed C57BL6/J × 129 x CD1 genetic background and were housed at the serum pathogen free (SPF) barrier area of the CNIO. Mice were treated in accordance with the Spanish Laws and the Guidelines for Human Endpoints for animals used in Biomedical Research. Mice were observed daily and killed when they showed signs of morbidity or overt tumors.

### Micro-computed tomography (micro-CT)

For micro-CT, mice were anesthetized with a continuous flow of 1% to 3% isoflurane/oxygen mixture (2 L/min). Acquisitions were performed using a micro-CT scanner Argus-Vista (SEDECAL, Madrid, Spain) including the whole body in 2-bed position. Tomographic images were reconstructed using a 3D-FBP (filtered back projection) algorithm that produced 55 slices measuring 55 × 55 pixels each. The isotropic resolution of this instrument was 45 µm. The micro-CT image acquisition consisted of 400 projections collected in one full rotation of the gantry in approximately 10 min per bed position. The image acquisition was made without any contrast agent. The X-ray tube settings were 80 kV and 450 µA. For image analysis and quantification, 3D Slicer software was used. Tumor volumes were measured once per week by micro-CT, to determine accurately the tridimensional tumor mass. The investigators were blinded during the entire in vivo experiment. Micro-CT measurements were performed in all cases with no information about the genotype or treatment of every mouse tested. The potential effects of miR-203 on metastasis incidence in the lungs were also analyzed by micro-CT throughout the three in vivo experiments.

### Mammary gland-derived organoids culture

*Tg (MMTV-PyMT); miR-203 (*+ */* +*)* and *Tg (MMTV-PyMT); miR-203 (KI/KI)* mice (treated or not with Dox in vivo, as indicated in the text) were euthanized, and tumors were extracted. Two random pieces were snap frozen and stored at − 80 °C; two random pieces were fixed in formalin for histopathology and immunohistochemistry analysis and the remainder was processed for the isolation of viable cells. The remaining tissue was minced, washed with 10 mL AdDF +  +  + (Advanced DMEM/F12 containing 1 × Glutamax, 10 mM HEPES, and antibiotics) and digested in 10 mL BC organoid expansion medium: 10% homemade R-Spondin 1 conditioned medium; 5 nM neuregulin 1 (Peprotech 100-03); 5 ng/mL FGF7 (Peprotech 100-19); 20 ng/mL FGF10 (Peprotech 100-26); 5 ng/mL EGF (Peprotech AF-100-15); 100 ng/mL Noggin (Peprotech 120-10C); 500 nM A83-01 (Tocris 2939); 5 µm Y-27632 (Abmole); 500 nM SB202190 (Sigma S7067); 1X B27 supplement (Gibco 17504-44); 1,25 mM N-Acetylcysteine (Sigma A9165); 5 mM nicotinamide (Sigma N0636); 1X Glutamax (Invitrogen 12634-034); 10 mM HEPES (Invitrogen 15630-056); 100U/mL Penicillin/Streptomycin (Invitrogen 15140-122); Primocin (Invitrogen Ant-pm-1); and Advanced DMEM/F12 (Invitrogen 12634-034), containing 1–2 mg/mL collagenase (Sigma, C9407). Digestion was performed on an orbital shaker at 37 °C for 1–2 h. The digested tissue suspension was sequentially sheared using 10 mL and 5 mL plastic and flamed glass Pasteur pipettes. After every shearing step the suspension was strained over a 100 μm filter with retained tissue pieces entering a subsequent shearing step with ∼10 mL AdDF +  +  + . 2% FCS were added to the strained suspension before centrifugation at 400 rcf. The pellet was resuspended in 10 mL AdDF +  +  + and centrifuged again at 400 rcf. In case of a visible red pellet, erythrocytes were lysed in 2 mL red blood cell lysis buffer (Roche, 11814389001) for 5 min at room temperature before the addition of 10 mL AdDF +  +  + and centrifugation at 400 rcf. The pellet was resuspended in 10 mg/mL cold Cultrex growth factor reduced BME type 2 (Trevigen, 3533-010-02), and 40 μL drops of BME-cell suspension were allowed to solidify on pre-warmed 24-well suspension culture plates (Greiner, M9312) at 37 °C for 20 min. Upon completed gelation, 400 μL of BC organoid expansion medium was added to each well and plates transferred to humidified 37 °C / 5% CO_2_ incubators. Medium was changed every 4 days, and organoids were passaged every week: Organoids were resuspended in 2 mL cold AdDF +  +  + and mechanically sheared through flamed glass Pasteur pipettes. When necessary, very dense organoids were dissociated by resuspension in 2 mL TrypLE Express (Invitrogen, 12605036), incubation for 1–5 min at room temperature, and mechanical shearing through flamed glass Pasteur pipettes. Following the addition of 10 mL AdDF +  +  + and centrifugation at 300 rcf. or 400 rcf., respectively, organoid fragments were resuspended in cold BME and reseeded as above at ratios (1:1 to 1:6) allowing the formation of new organoids. Single cell suspensions were initially seeded at high density and reseeded at a lower density after ∼1 week. In order to prevent misidentification and/or cross-contamination of BC organoids, we cultured every line physically separate. All organoid lines were frequently tested and resulted in all cases negative in the MycoAlert mycoplasma detection kit (Lonza, LT07-318). For epithelial differentiation, we used the media defined by Lonza (MEGM Mammary Epithelial Cell Growth Medium and Bullekit). Basically, this media has been optimized for the growth of mammary epithelial cells in a serum-free environment and includes BPE, hEGF, insulin, hydrocortisone and GA-1000 (Lonza CC-3150). FGF2 treatment (2 nM; Sigma) was used to induce mammary branching as published before [56]. For inducing transient miR-203 over-expression, ColA1(miR-203/miR-203); Rosa26(rtTA/rtTA) organoid cultures were treated with Dox (1 µg /mL; Invitrogen) during 5 days. After that, Dox withdrawal was standardized for the cultures during following several passages (usually 2 weeks) unless other time points are indicated in the text. In this inducible system, we always test that insert expression is uniquely dependent on Dox and becomes absolutely undetectable after Dox withdrawal. As a control of the treatment itself, Dox was also added and tested in wild-type organoids.

### Patient-derived organoids generation and culture

For this study, breast cancer patients (with BIRAD 4C-5-6) from Hospital *12 de Octubre* (Madrid, Spain) donated one cylinder of the first core-needle tumor biopsy, prior diagnosis. To guarantee the protection of patients enrolled in this study, we have strictly followed the hospital guidance, the local regulations, the “Declaration of Helsinki” and the Guidelines of good clinical practice from the “International Conference on Harmonization” ICH E6 (R2), effective from June 14, 2017. The technical protocols for patient-derived sample collection and processing and any additional material delivered to the patient (such as Patient Information Sheets or the Informed Consent Document) were carefully evaluated and approved by the corresponding Clinical Research Ethics Committee, in accordance with national legislation. Tumor samples were immediately processed in our laboratory for organoid culture generation, as described above. We were able to maintain patient-derived organoid cultures for three or four passages, and the experiments were always performed at passage one. After 7 days of culture establishment and organoid amplification, patient-derived 3D cultures were transiently transfected with miR-203 mimics, followed by miR-203 withdrawal for three additional weeks. Hsa-miR-203 mimics were purchased from Sigma-Aldrich (MISSION microRNA mimics), and transient transfection was performed using Lipofectamine 2000 (Sigma), following manufacturer’s instructions. Since then, cultures were carefully evaluated under the bright-field microscope for quantification of organoid number and size, complexity, formation of 2D projections, and finally, immunofluorescence was performed at the end of the experiment (three weeks after the miR-203 brief exposure).

### Immunofluorescence and immunohistochemistry

Organoids were fixed in 4% paraformaldehyde for at least 15 min, permeabilized using PBS 0.1% Triton X-100 for 15 min and blocked in BSA for 1 h at room temperature. Primary antibody incubation was performed overnight at 4ºC in all cases, followed by secondary antibody incubation for 1 h at room temperature. Nuclear staining was included in the last PBS wash, using Hoechst or DAPI. Primary antibodies used in this study were against CK8/18 (rabbit monoclonal EP17/EP30, Dako, IR094), CK14 (rabbit polyclonal AF64, Covance, PRB-155P) and E-cadherin (mouse monoclonal 36, BD Bioscience, 610182) for mouse-derived samples and CK8/18 (rat monoclonal, DSHB, 531826), CK14 (rabbit monoclonal, Abcam, ab181595) and vimentin (mouse monoclonal RV202, BD Pharmingen, 550513) for patient-derived samples. Cells were examined under a Leica SP5 microscope equipped with white light laser and hybrid detection.

For immunohistochemistry, tissue samples were fixed in 10% neutral buffered formalin (4% formaldehyde in solution), paraffin-embedded and cut at 3 µm, mounted in superfrost®plus slides and dried overnight. Consecutive sections were stained with hematoxylin and eosin (H&E) or subjected to immunohistochemistry using automated immunostaining platforms (Ventana Discovery XT, Roche or Autostainer Plus Link 48). Antigen retrieval was first performed with high or low pH buffer depending on the primary antibody (CC1m, Roche or low pH antigen retrieval buffer, Dako), endogenous peroxidase was blocked (peroxide hydrogen at 3%), and slides were incubated with primary antibodies against Ki67 (rabbit monoclonal D3B5, Cell Signalling Technology, 12202), cleaved Caspase 3 (rabbit, Cell Signalling Technology, 9661), CK5 (rabbit polyclonal AF 138, Covance, PRB-160P), SOX-10 (goat polyclonal N20, Santa Cruz Biotechnology, sc-17342), CD44 (rabbit polyclonal, Abcam, ab157107), H3K27me3 (rabbit monoclonal C36B11, Cell Signalling Technology, 9733), prolactin (rabbit polyclonal, Dako, A0569), progesterone receptor (rabbit monoclonal SP2, Thermo Scientific, RM-9102-R7), NeuN (mouse monoclonal A60, Millipore, MAB377), E-cadherin (mouse monoclonal 36, BD Bioscience, 610182), Aldh1/2 (mouse monoclonal H-8, Santa Cruz Biotechnology, sc-166362), CK8/18 (rabbit monoclonal EP17/EP30, Dako, IR094), CK14 (rabbit polyclonal AF64, Covance, PRB-155P), smooth muscle actin (mouse monoclonal 1A4, Dako, IR611), estrogen receptor alpha (rabbit polyclonal, Santa Cruz Biotechnology, sc-542).

Secondary antibodies were conjugated with horseradish peroxidase (OmniRabbit, Ventana, Roche), and the immunohistochemical reaction was developed using 3,30-diaminobenzidine tetrahydrochloride (DAB) as a chromogen (Chromomap DAB, Ventana, Roche or DAB solution, Dako) and nuclei were counterstained with Carazzi’s hematoxylin. Finally, the slides were dehydrated, cleared and mounted with a permanent mounting medium for microscopic evaluation. The images were acquired with a slide scanner (AxioScan Z1, Zeiss). Images were captured and quantified using the Zen Software (Zeiss).

### Analysis of mRNA levels, RNA sequencing

RNA/microRNA was extracted from organoids samples with TRIzol (Invitrogen) or by using the miRVana isolation kit (Thermo Fisher), following the manufacturer’s recommendations and after the dissociation of Matrigel/BME from the cultures by using the Cell Recovery Solution (Corning), following the manufacturer’s protocols. For reverse transcription of microRNAs, we used the TaqMan small RNA assay (4366596), including the specific oligonucleotides for mmu-miR-203-5p and 3p (002580 and 000507), miR-16 and the housekeeping RNAs sno-202 or sno-142. Conditions for miRNA amplification were as follows: 30 min at 16ºC; 30 min at 42ºC and a final step of 5 min at 85ºC. Quantitative real-time PCR was then performed using the TaqMan Universal PCR Master Mix (434437) following the manufacturer’s instructions in an ABI PRISM 7700 Thermocycler (Applied Biosystems).

For RNAseq, total RNA was extracted using the miRVana miRNA isolation kit (Thermo Fisher), following the manufacturer’s recommendations. Between 0.8 and 1 µg of total RNA were extracted from organoids after dissociating the Matrigel/BME from the cultures (as indicated above). RIN (RNA integrity number) numbers were always in the range of 9 to 10 (Agilent 2100 Bioanalyzer). 250 ng of total RNA samples was used. Average sample RNA integrity number was 9.1 (range 8.2–9.8) when assayed on an Agilent 2100 Bioanalyzer. Sequencing libraries were prepared with the “QuantSeq 3’ mRNA-Seq Library Prep Kit (FWD) for Illumina” (Lexogen, Cat. No. 015) by following manufacturer instructions. This kit generates directional libraries stranded in the sense orientation, the read1 (the only read in single read format) has the sense orientation. Library generation is initiated by reverse transcription with oligo dT priming, and a second strand synthesis is performed from random primers by a DNA polymerase. Primers from both steps contain Illumina-compatible sequences. Libraries were completed by PCR, applied to an Illumina flow cell for cluster generation and sequenced on an Illumina HiSeq 2500 with v4 Chemistry by following manufacturer’s protocols. Read adapters and polyA tails were removed with bbduk.sh (https://sourceforge.net/projects/bbmap/), following the Lexogen recommendations. Processed reads were analyzed with the nextpresso pipeline [75], as follows: Sequencing quality was checked with FastQC v0.11.7 (http://www.bioinformatics.babraham.ac.uk/projects/fastqc/). Reads were aligned to the mouse reference genome (GRCm38) with TopHat-2.0.10 [76] using Bowtie 1.0.0 [77] and Samtools 0.1.19 [78] (library-type fr-secondstrand in TopHat), allowing two mismatches and twenty multihits. Read counts were obtained with HTSeq-count v0.6.1 [79] (stranded = yes), using the mouse gene annotation from GENCODE (gencode.vM20.GRCm38.Ensembl95). Differential expression was performed with DESeq2 [80], using a 0.05 FDR. GSEA Pre-ranked [81] was used to perform gene set enrichment analysis for several gene signatures on a pre-ranked gene list, setting 1000 gene set permutations. Only those gene sets with significant enrichment levels (FDR q-value < 0.25) were considered.

## Statistics

Samples (organoids or mice) were allocated to their experimental groups according to their pre-determined type, and therefore, there was no randomization. Investigators were blinded to the experimental groups in all cases. Normal distribution and variance was confirmed for all samples and experiments performed. Based on this, we used the Student’s t test (two-tailed, unpaired) to estimate statistical significance when two groups were compared. Whenever necessary, we used One-way ANOVA to compare variances across the means of three different groups. Statistical analysis was performed using Prism (GraphPad Software, La Jolla, CA). All the experiments presented in this work were performed at least 3 times (between 3 and 10 independent biological replicates, except for patient-derived samples, where 3 technical replicates from 2 independent biopsies were included in the analyses). Measurements of IHC/IF mean intensity were performed by the standard intensity function in the open source Fiji software (ImageJ) (http://fiji.sc/Fiji). In those cases, six different fields from three independent tumor samples or organoid cultures were analyzed. For organoid shape distribution, five fields from three independent organoid cultures were quantified.

### Supplementary Information

**Additional file 1**. Supplementary Figures S1–S4.

**Additional file 2**. Supplementary table, extended excel data for Supplementary Figure S4.

## Data Availability

RNAseq data have been deposited in the GEO repository under accession number GSE202831.
