# Supplementary material for: miR-203 drives breast cancer cell differentiation
Source: Breast Cancer Res. 2023 Aug 4;25:91. doi: 10.1186/s13058-023-01690-9 (PMC10401798; doi:10.1186/s13058-023-01690-9)
Supplement: Supplementary file 1 — Additional file 1. Supplementary Figures S1–S4. [file 13058_2023_1690_MOESM1_ESM.pdf]

miR-203 drives breast cancer cell differentiation

(Supplementary Information)

Nuria G. Martínez-Illescas,[1,2,3] Silvia Leal,[4] Patricia González,[5] Osvaldo Graña-Castro,[6,7] Juan José Muñoz-Oliveira,[8] Alfonso Cortés-Peña,[8] María Gómez-Gil,[5] Zaira Vega,[5] Verónica Neva,[5] Andrea Romero,[5] Miguel Quintela-Fandino,[9] Eva Ciruelos,[10,2] Consuelo Sanz,[10,2] Sofía Aragón,[10,2] Leisy Sotolongo,[10,2] Sara Jiménez,[10,2] Eduardo Caleiras,[5] Francisca Mulero,[4] Cristina Sánchez,[1,2] Marcos Malumbres,*[3,11,12] María Salazar-Roa*[1,2,3]*

The authors declare no competing financial interests.

<u>Supplementary Information</u>

Supplementary Figure S1. Assessment of proliferation and apoptosis in PyMT mammary tumors at tumor onset and endpoint, treated or not with miR-203.

Supplementary Figure S2. miR-203-promoted morphological changes on mammary tumor organoids compared to those triggered by other well-known differentiation stimuli.

Supplementary Figure S3. RNA sequencing of organoid samples, derived from healthy or tumor tissue, and exposed *in vitro* to miR-203.

Supplementary Figure S4. Analysis of specific mRNA profiles for basal cells, development, cell migration, metabolism and cell cycle in organoid samples, derived from healthy or tumor tissue, and exposed *in vitro* to miR-203.

Supplementary Table 1 (extended excel data for Supplementary Figure S4). Extended data of the RNA sequencing experiments, displayed in Supplementary Figure 4B. Signatures analyzed and herein reported include: “organ and cell development”, “cell migration and motility”, “metabolism” and “cell cycle”.

Supplementary Fig. 1

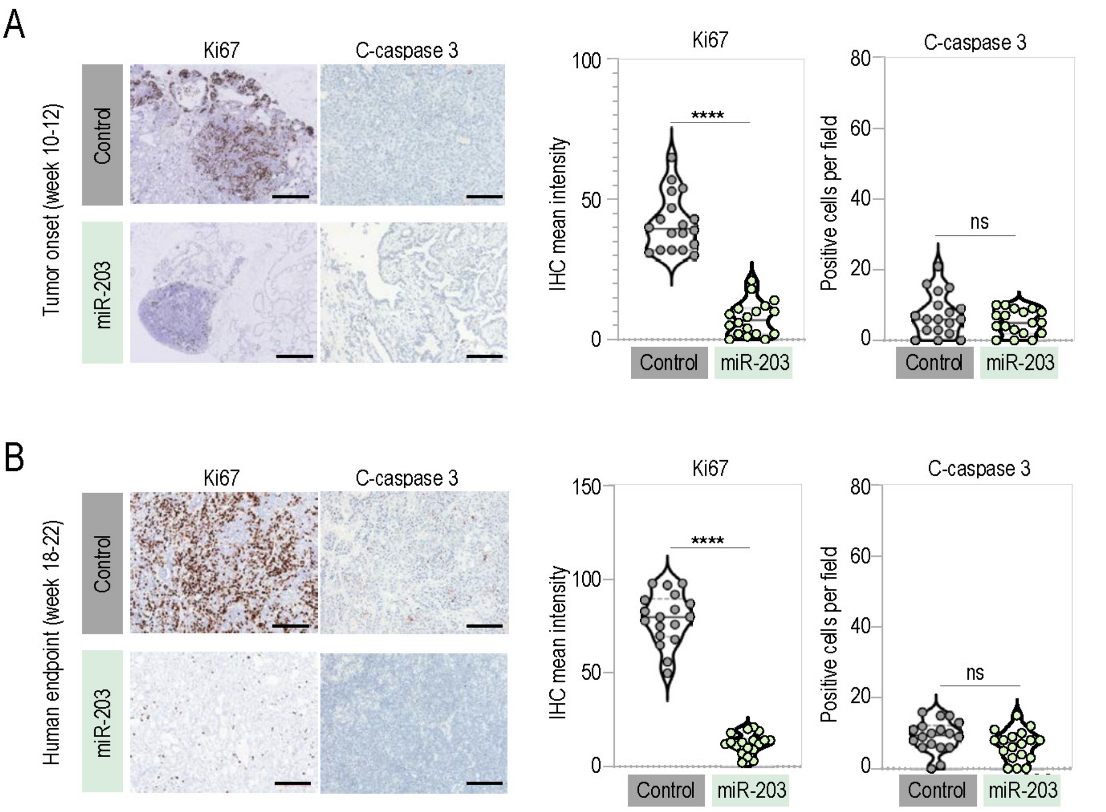

Supplementary Fig. 1. Assessment of proliferation and apoptosis in PyMT mammary tumors at tumor onset and endpoint, treated or not with miR-203. Left panels, Illustrative immune-histopathological analysis of representative tumors (from *miR-203 wild-type or miR-203 knock-in; PyMT* mice exposed to Dox *in vivo*) and analyzed at two different time points: tumor onset (week 10-12, when tumors are not generally noticed by micro-CT yet, but identified by histopathology analysis, panel A) or the experimental human endpoint (week 18-22; panel B). Ki-67 (as proliferation marker) and cleaved caspase 3 (as apoptosis marker) staining are shown. Right panels, Violin plots showing the quantification of Ki-67 or Cleaved caspase 3 staining (six different fields from three independent tumor samples were analyzed). Scale bar, 500 μm. ****p<0.0001; *n.s.* not statistically different (Student´s t-test).

Supplementary Fig. 2

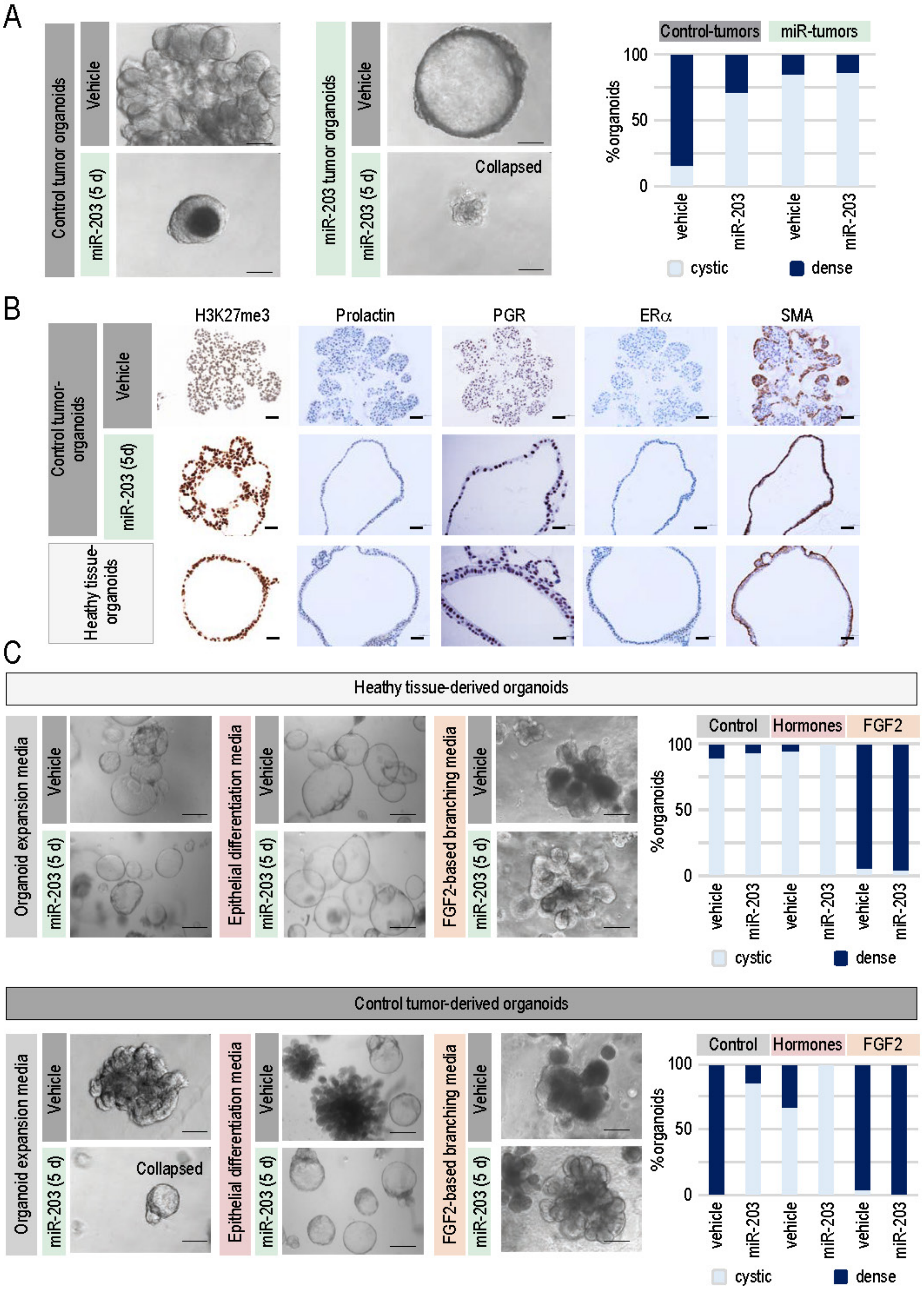

Supplementary Fig. 2. miR-203-promoted morphological changes on mammary tumor organoids compared to those triggered by other well-known differentiation stimuli. A, Left panel, Representative bright-field images of tumor-derived organoids (tumors from *miR-203 knock-in; PyMT* mice treated *in vivo* either with vehicle or Dox), exposed *in vitro* to vehicle or miR-203 (Dox) during 5 days and followed by miR-203 withdrawal for 2 more weeks (indicated as "miR-203 5d" in the figure). Example of collapsing organoids is shown, in the miR-203 exposed cultures. Right panel, quantification of the percentage of organoids exhibiting dense *versus* cystic (luminal-like) morphology in every condition tested. B, Illustrative images of IHC staining for H3K27me3, prolactin, progesterone receptor (PGR), estrogen receptor alpha (ERα) and smooth muscle actin (SMA) in control tumor-derived organoids (upper panels), control tumor-derived organoids treated *in vitro* with miR-203 during 5 days (middle panels) and healthy mammary gland-derived organoids (bottom panels). Quantifications of these staining are shown in Figure 5E. C, Representative bright-filed images of healthy tissue-derived organoids (upper panels) or control tumor-derived organoids (bottom panels), exposed *in vitro* during 2 weeks to the indicated treatments: left panels, organoids cultured on basic expansion media, and treated with vehicle or miR-203 during the first 5 days; middle panels, organoids cultured on epithelial differentiation media (consisting on prolactin, insulin, epidermal growth factor, hydrocortisone, bovine pituitary extract and gentamicin/amphotericin B), and treated with vehicle or miR-203 during the first 5 days; right panels, organoids cultured on FGF2-based branching induction media, and treated with vehicle or miR-203 during the first 5 days. Quantification of the percentage of organoids exhibiting cystic or dense shapes respect to the total number of organoids is shown for each condition tested. In A, C: scale bar, 100 μm; in B: scale bar, 500 μm.

Supplementary Fig. 3

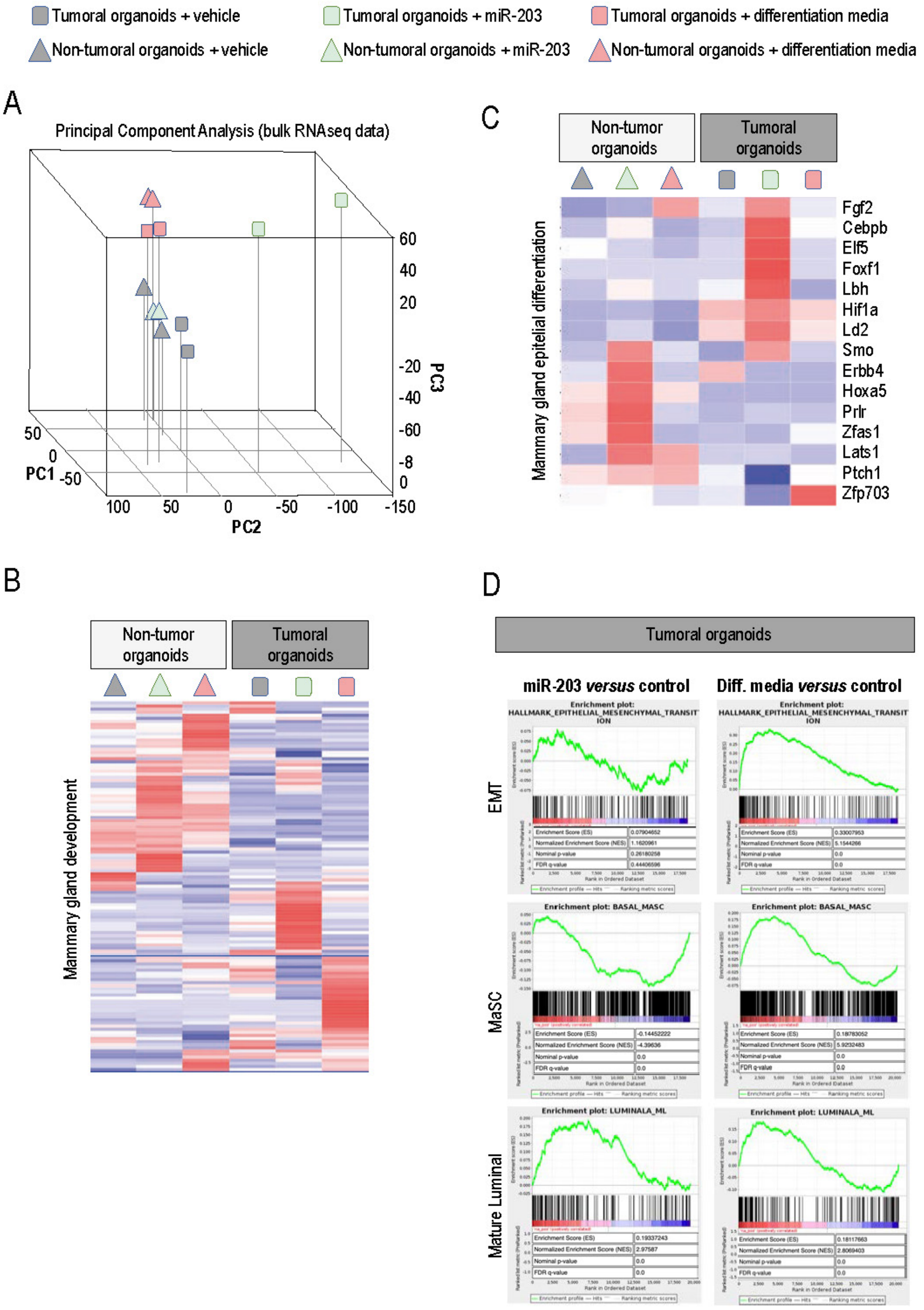

Supplementary Fig. 3. RNA sequencing of organoid samples, derived from healthy or tumor tissue, and exposed *in vitro* to miR-203. A, Principal Component Analysis of RNA sequencing performed on the samples indicated in the figure: non-tumor (triangles) or tumor (squares) mammary-gland-derived organoids, exposed to either vehicle (in grey), miR-203 for 5 days *in vitro* (in green) or differentiation media (consisting on prolactin, insulin, hEGF, hydrocortisone, BPE (bovine pituitary extract) and gentamicin/amphotericin B; in pink). The color and shapes code will be maintained throughout the figure, for clarity. B, C, Heatmaps showing the Gene Ontology signature for mammary gland development (B) and epithelial differentiation (C) in all the samples tested by RNA sequencing, as indicated (color and shape code as in A). D, Enrichment GSEA plots showing the induction of gene signatures characteristic of Epithelial-to-Mesenchymal Transition (EMT), Mammary Stem Cells (MaSC) and Mature Luminal cells in the comparisons indicated: miR-203-treated *versus* control tumor organoids (left panels) and differentiation media-cultured *versus* control tumor organoids (right panels). Insets in the GSEA plots include information about the enrichment score, normalized enrichment score, nominal p-value and FDR q-value for each comparison.

Supplementary Fig. 4

Tumoral organoids	miR-203 *versus* control: Significantly down-regulated genes

A

ARCHS4 Tissues data base

term	p-value	q-value	overlap_genes
Basal cells	9.741974e-20	3.507111e-18	FOXE1, FAM57A, GALNT18, TNC, FOXI1, CALML3, TFCP2L1, PRSS22, ESPN, DMKN, AQP3, WFDC5, PTPRF, NKPD1, HK2, GJA1, CYP26B1, NIPAL1, TRIM29, SLC16A3, KRT6A, BOK, KRTDAP, CERS3, ELOVL4, IL18, KRT5, OVOL1, OSMR, HSPG2, SERPINB8, PGF, EPN3, PROCR, SLPI, ELF3, ADGRF4, MALL, DDIT4, LY6D, FSCN1, KCTD11, DSG3, IVL, HBEGF, SEMA3B, IL20RB, LYPD3, SBSN, KLK8, CST6, NDRG1, BARX2, KLK7, KLK6, MTHFD1L, SH3BP1, STC2, P2RY1, PLEK2, SFN, LY6G6C, TSPAN1, NGFR, WNT10A, JUP, CAVIN3, KLK13, G0S2, EPHX3, KLF4, KLK10, KLK11, VEGFA, GJB2, KRT17, NLRP10, P4HA2, REEP4, KRT14, BCAR1

PanglaoDB Augmented 2021

term	p-value	q-value	overlap_genes
Basal Cells	9.941465e-14	4.473659e-12	[BNIP3, KRT5, PRSS22, CST6, GJB2, SLPI, KRT17, TRIM29, ADGRF4, MALL, KRT14, LY6D, PLEK2, SFN, S100A14, SLC16A3, TSPAN1, KRT6A]

B

Organ & Cell development

Term	GO	Count genes	%	P-value	Benjamini
Animal organ development	GO:0048513	71	30,5	2,8E-06	1,3E-03
Cell development	GO:0048468	37	15,9	5,7E-02	7,2E-01

Cell migration & Motility

Cell Migration	GO:0016477	33	14,2	1,3E-04	2,3E-02
Regulation of cell migration	GO:0030334	24	10,3	2,8E-04	3,8E-02
Regulation of cell motility	GO:2000145	24	10,3	6,4E-04	5,7E-02
Positive regulation of cell migration	GO:0030335	15	6,4	3,6E-03	1,6E-01

Metabolism

Protein metabolic process	GO:0019538	74	31,8	1,7E-03	1,1E-01
Negative regulation of protein metabolic process	GO:0051248	26	11,2	1,4E-03	9,8E-02
Regulation of protein metabolic process	GO:0051246	49	21,0	1,6E-03	1,0E-01
negative regulation of cellular metabolic process	GO:0031324	46	19,7	2,3E-03	1,2E-01

Cell cycle

Cell Cycle	GO:0007049	13	5,6	6,7E-02	1,0E+00

Supplementary Fig. 4. **Analysis of specific mRNA profiles for basal cells, development, cell migration, metabolism and cell cycle in organoid samples, derived from healthy or tumor tissue, and exposed *in vitro* to miR-203**. A, "Basal cell" signatures differentially down-regulated in miR-203-exposed tumor organoids *versus* their corresponding control counterparts, according to Enrichr (https://maayanlab.cloud/Enrichr/) (1) in two different data bases: ARCHS4 and PanglaoDB. B, Gene Ontology (GO) Signatures for "organ and cell development", "cell migration and motility", "metabolism" and "cell cycle" differentially down-regulated in miR-203-exposed tumor organoids *versus* their corresponding control counterparts, as defined by "The Database for Annotation, Visualization and Integrated Discovery" DAVID (https://david.ncifcrf.gov/) (2). All the genes recognized as significantly down-regulated in miR-203 treated *versus* control tumor samples and clustered in any of the signatures herein included are listed in the Figure and in the Supplementary Table 1.

Supplementary Table 1 (extended excel data for Supplementary Figure S4). Extended data of the RNA sequencing experiments, displayed in Supplementary Figure 4B. Signatures analyzed and herein reported include: "organ and cell development", "cell migration and motility", "metabolism" and "cell cycle".

References in Supplementary Figures:

1. Xie Z, Bailey A, Kuleshov MV, Clarke DJB, Evangelista JE, Jenkins SL, et al. Gene Set Knowledge Discovery with Enrichr. Curr Protoc. 2021;1(3):e90.

2. Sherman BT, Hao M, Qiu J, Jiao X, Baseler MW, Lane HC, et al. DAVID: a web server for functional enrichment analysis and functional annotation of gene lists (2021 update). Nucleic Acids Res. 2022.
